# Prevalence and trend of atrial fibrillation and its associated risk factors among the population from nationwide health check-up centers in China, 2012–2017

**DOI:** 10.3389/fcvm.2023.1151575

**Published:** 2023-05-31

**Authors:** Tao Sun, Mao Ye, Fang Lei, Juan-Juan Qin, Ye-Mao Liu, Ze Chen, Ming-Ming Chen, Chengzhang Yang, Peng Zhang, Yan-Xiao Ji, Xiao-Jing Zhang, Zhi-Gang She, Jingjing Cai, Zhao-Xia Jin, Hongliang Li

**Affiliations:** ^1^ Department of Cardiology, Renmin Hospital of Wuhan University, Wuhan, China; ^2^ Institute of Model Animal, Wuhan University, Wuhan, China; ^3^ Department of Cardiology, Huanggang Central Hospital of Yangtze University, Huanggang, China; ^4^ Translation Medicine Research Center of Yangtze University, Huanggang, China; ^5^ School of Basic Medical Science, Wuhan University, Wuhan, China; ^6^ Department of Cardiology, Zhongnan Hospital of Wuhan University, Wuhan, China; ^7^ Department of Cardiology, The Third Xiangya Hospital, Central South University, Changsha, China; ^8^ Medical Science Research Center, Zhongnan Hospital of Wuhan University, Wuhan, China

**Keywords:** atrial fibrillation, prevalence, trend, risk factors, China

## Abstract

**Background:**

Atrial fibrillation (AF) is the most prevalent cardiac arrhythmia, which poses huge disease burdens in China. A study was conducted to systematically analyze the recent prevalence trend of AF and age-related disparities in AF risk among the nationwide healthy check-up population.

**Method:**

We conducted a nationwide cross-sectional study involving 3,049,178 individuals ≥35 years from health check-up centers to explore the prevalence and trend of AF by age, sex, and region from 2012 to 2017. Additionally, we analyzed risk factors associated with AF among the overall population and different age groups via the Boruta algorithm, the LASSO regression, and the Logistic regression.

**Result:**

The age-, sex-. and regional-standardized prevalence of AF kept stable between 0.4%–0.45% among national physical examination individuals from 2012 to 2017. However, the prevalence of AF showed an undesirable upward trend in the 35–44-year age group (annual percentage changes (APC): 15.16 [95%CI: 6.42,24.62]). With increasing age, the risk of AF associated with the overweight or obesity gradually exceeds that associated with diabetes and hypertension. In addition to traditional leading risk factors such as age≥65 and coronary heart disease, elevated uric acid and impaired renal function were tightly correlated with AF in the population.

**Conclusion:**

The significant rise in the prevalence of AF in the 35–44 age group reminds us that in addition to the elderly (the high-risk group), younger people seem to be in more urgent need of attention. Age-related disparities in AF risk also exist. This updated information may provide references for the national prevention and control of AF.

## 1. Introduction

Atrial fibrillation (AF) is the most prevalent cardiac arrhythmia contributing to adverse cardiovascular events, including heart failure, systemic embolism, and stroke (1). The Global Burden of Disease Study 2017 revealed that the prevalence of AF is 0.48% worldwide with an increased number of patients (2, 3). The prevalence and cost of AF in China are also growing rapidly due to aging, economic growth, and the increased prevalence of its related risk factors. Previous studies based on representative sampling showed that the standardized prevalence of AF in the Chinese population was 0.65%–2.31% (4–6). Recently, a nationwide study with a multistage stratified sampling of 114,039 people reported that the prevalence of AF in the Chinese adult population was 1.6%, a 146% increase compared to two decades ago (7). Reducing the disease burden of AF in China represents a huge challenge. For more individualized and effective control of AF, comprehensively and granularly understanding the trend in the prevalence of AF is required. However, these cross-sectional studies are limited in reflecting the changing trend due to data from a single cross-section. Thus, it is greatly necessary to systematically study the recent changing trend of AF in different dimensions (e.g., sexes, age groups, and regions). Meanwhile, we could explore the potential risk factors for AF in the general population and age subgroups by utilizing the comprehensive biochemical indicators included in the physical examinations.

Over 500 million people with diverse socioeconomic backgrounds (public service employees, workers, self-employed persons, farmers, and others) participate in health examinations in China each year (8). The majority of enterprises and institutions offer health examinations to their employees as a health benefit. Chronic AF is often asymptomatic. However, patients with unrecognized AF may present with thromboembolic consequences or tachycardia-mediated cardiomyopathy. Therefore, the nationwide health check-up routine provides great opportunities for early diagnosis and intervention of AF and for identifying potential risk factors contributing to its prevalence.

In this study, we aimed to analyze the prevalence and trend of AF by sex, region, and age and associated risk factors among the health check-up population between 2012 and 2017 using the largest cross-sectional database of 3,049,178 individuals from 27 health management centers in China. These findings would be an essential reference for developing prevention and control strategies.

## 2. Materials and methods

### 2.1. Study population

In this cross-sectional study, a total of 3,049,178 participants were included from 27 Health Check-up Centers in 15 provinces or municipalities in 2012 and 2017, which were selected according to geographic location, economic level, and available data. These centers are located in provinces or municipalities that span the north and south of the Chinese mainland (latitudes 20.2° N to 53.3°N). Among them, 12 centers with 1,314,043 participants are located in northern China, and 15 centers with 1,735,135 participants are located in southern China (Supplementary Table S1). Meanwhile, these provinces or municipalities contain three different (low, medium, and high) economic levels, divided according to the 2015 (the middle year of 2012–2017) GDP per capita of each province. High-economic regions are Beijing, Guangdong, Inner Mongolia, Liaoning, Shandong, and Tianjin; Medium-economic regions are Chongqing, Hubei, Hunan, Hebei, and Jilin; and regions with low-economic levels are Gansu, Henan, Jiangxi, and Shanxi. All the participants were aged 35 years and older without repeated measurement records. The study was approved by the central ethics board of Renmin Hospital. Individual identification data was removed, and only anonymous information was kept during the study. The ethics committees of each hospital waived patient informed consent.

### 2.2. Data collection and measurement

Each center was staffed by experienced medical teams, and all individuals had undergone medical history collection, comprehensive anthropometric measurements, and clinical examinations. The medical history collection was obtained face-to-face and recorded by professional physicians, including living habits, medical history, family medical history, and current medication. According to standard protocols, anthropometric measurements such as height, weight, waist circumference, hip circumference, and systolic and diastolic blood pressures (SBP and DBP) were collected. Body mass index (BMI) was calculated as weight divided by the square of height (kg/m^2^). Twelve-lead ECGs for all participants were recorded and interpreted by trained cardiologists. In addition, after an overnight fast of ≥8 h, fasting blood samples of participants were collected in the morning for routine blood tests and biochemical tests.

### 2.3. Diagnosis of AF

According to the ACC/AHA/ESC 2006 guidelines and the 2016 ESC guidelines, the diagnosis of AF was based on medical history and a 12-lead ECG as follows: (1) Irregular rhythm; (2) No discernible *P* waves; (3) Absence of an isoelectric baseline; (4) Variable ventricular rate (9, 10).

### 2.4. Definition of risk factors

According to the WHO criteria for Asians, a BMI ≥ 24 Kg/m^2^ was defined as overweight or obesity (11). Following China's 2018 guidelines, hypertension is identified as clinic SBP ≥140 mmHg and/or DBP ≥90 mmHg, a self-reported history of hypertension, or the use of anti-hypertensive medications (12). Type 2 diabetes mellitus (T2DM) was diagnosed based on guidelines for preventing and controlling T2DM in China and personal history (13). Coronary heart disease (CHD) was defined as self-reported CHD. Dyslipidemia was diagnosed if participants met either of the following criteria: (1) Diagnostic criteria for dyslipidemia in the guidelines for the prevention and treatment of dyslipidemia in Chinese adults (triglycerides (TG) ≥ 2.30 mmol/L or total cholesterol (TC) ≥ 6.2 mmol/L or Low-density lipoprotein cholesterol (LDL-C) ≥ 4.9 mmol/L or high-density lipoprotein cholesterol (HDL-C) < 1.0 mmol/L) (14); (2) self-reported dyslipidemia or use of lipid-lowering drugs. Elevated uric acid (UA) was defined as a blood uric acid level > 420 μmol/L in males and >360 μmol/L in females (15). Elevated creatinine for males was defined as a serum creatinine level > 93 μmol/L in those younger than 60 years old and > 109 μmol/L in those older than 60 years old; and for females was defined as a serum creatinine level > 69 μmol/L in those younger than 60 years old and >83 μmol/L in those older than 60 years old (16). Elevated blood urea nitrogen (BUN) was defined as a BUN level > 7.1 mmol/L (17). According to the guidelines, elevated TG, TC, and LDL-C were defined as a TG level ≥ 1.7 mmol/L, a TC level ≥ 5.2 mmol/L, an LDL-C level ≥ 3.4 mmol/L, respectively; and decreased HDL-C was defined as an HDL-C level < 1 mmol/L (18).

### 2.5. Statistical analysis

The basic characteristics of the participants were presented through descriptive statistics. Continuous variables with a normal distribution were described by mean and standard deviation (SD), and continuous variables with non-normal distribution were described by median and interquartile range. Categorical variables were described by numbers and percentages (%). Intergroup differences between groups were compared using Student's *t*-tests (normally distributed) and Wilcoxon rank-sum tests (non-normally distributed) for continuous variables. Fisher's exact test or *χ*^2^ test was used to compare differences for categorical variables. The age-sex-and regional-standardized prevalence of AF was calculated with specific weights based on China's 2010 National Population Census; the prevalence and trend of AF in different age groups, regions, and disease subgroups were also analyzed. We calculated the annual percent change (APC) by the Joinpoint regression to quantify the time trends of AF prevalence (19, 20). And the corresponding standard errors were obtained by the width of 95% UI divided by 1.96  ×  2. If the APC was significantly different from 0, an increasing (worsening) or decreasing (improving) trend was defined; if no difference from zero was noted, a stable or level trend was defined.

To comprehensively explore the relative risk factors of AF, we have also extensively included blood cells, blood lipids, and other indicators related to the heart, liver, kidneys, etc. The small number of missing values was filled by a random forest model, and more details about the method of imputation were reported in previous studies (21). The missing rates of variables included in the analysis are shown in Supplementary Table S2.

We applied Boruta analysis and the Least Absolute Shrinkage and Selection Operator (LASSO) regression to select the most valuable risk factors and used logistic regression for further analysis. The main idea of Boruta is to compare the importance of the real predictor variables with those of random so-called shadow variables using statistical testing and multiple runs of random forest, which provide an unbiased grading of the prognostic importance of all variables (22, 23). Then, variables performing better than the maximum random variable importance are defined as “confirmed”, variables performing worse are “rejected”, and variables that cannot be confirmed or rejected are defined as “tentative”. The LASSO model is a shrinkage method within the least square method that enables to shrink estimation of least contributive variables towards zero (24). By LASSO regression, we can objectively select a subset of variables and eliminate potential collinearity (25). We selected the tuning parameter lambda (*λ*), which determines the coefficient shrinkage by 10-fold cross-validation. Then, we selected risk factors by the coefficients calculated by plugging in the minimum lambda (*λ*). Finally, we further conducted an analysis of selected risk factors by multivariate logistic regression. All the risk factors selected (age, sex, CHD, T2DM, hypertension, overweight or obesity, UA, BUN, creatinine, TC, TG, HDL-C, and ALT) were included in logistic regression for mutual adjustment. In addition, because of the relatively low prevalence of AF, the severe class imbalance between AF and non-AF groups will affect the reliability of Boruta, LASSO, and logistic regression results. Thus, the ROSE (Random Over Sampling Examples) package, a synthetic minority sampling technique that interpolates from minority classes while down sampling the majority class, is used to solve this problem (26, 27). Joinpoint regression and APC calculations were performed using the Joinpoint regression program (Version 4.9.0.0, Statistical Methodology and Applications Branch and Data Modeling Branch, National Cancer Institute, Bethesda, MD, USA). All other statistical analyses were conducted in R software (version 3.6.3); *p* values < 0.05 were considered statistically significant.

## 3. Result

### 3.1. Baseline characteristics of study participants

There were 3,049,178 individuals included from 2012–2017 in this cross-sectional study. The mean age was 51.16 (SD, 11.55) years, and 59.4% of participants were male. The mean BMI was 24.62 (SD, 3.28) kg/m^2^. The mean systolic and diastolic blood pressures were 127 (SD, 19) mmHg and 79 (SD, 12) mmHg, respectively. There were 848,374 (30.2%) individuals with hypertension, 248,334 (8.4%) with T2DM, 59,529 (2.0%) with CHD, 1,337,675 (56.0%) with overweight or obesity, and 1,173,927 (41.7%) with dyslipidemia (Table 1).

**Table 1:** Baseline characteristics of study participants.

Characteristics	Total	Total (*N* = 3,049,178)		
	(*N* = 3,049,178)	Non-AF	AF	*P*-value
		(*N* = 3,037,694)	(*N* = 11,484)	
**Clinical Characteristics**				
Age (year, mean (SD))	51.16 (11.55)	51.09 (11.50)	68.84 (12.22)	<0.001
Sex, Male (%)	1,811,433 (59.4)	1,803,126 (59.4)	8,307 (72.3)	<0.001
BMI (kg/m^2^, mean (SD))	24.62 (3.28)	24.61 (3.28)	25.66 (3.60)	<0.001
SBP (mmHg, mean (SD))	127 (19)	127 (19)	134 (20)	<0.001
DBP (mmHg, mean (SD))	79 (12)	79 (12)	82 (14)	<0.001
**Laboratory Examination**				
FBG (mmol/L, mean (SD))	5.57 (1.39)	5.56 (1.39)	5.95 (1.61)	<0.001
TC (mmol/L, mean (SD))	4.96 (1.01)	4.96 (1.01)	4.57 (1.01)	<0.001
TG (mmol/L, mean (SD))	1.74 (1.55)	1.74 (1.55)	1.44 (1.00)	<0.001
LDL-C (mmol/L, mean (SD))	2.78 (0.79)	2.78 (0.79)	2.57 (0.83)	<0.001
HDL-C (mmol/L, mean (SD))	1.37 (0.36)	1.37 (0.36)	1.32 (0.34)	<0.001
ALT (IU/L, mean (SD))	25.32 (22.12)	25.34 (22.14)	22.13 (15.51)	<0.001
AST (IU/L, mean (SD))	24.50 (14.55)	24.49 (14.56)	25.81 (11.03)	<0.001
BUN (mmol/L, mean (SD))	5.00 (1.44)	4.99 (1.44)	5.98 (1.91)	<0.001
UA (μmol/L, mean (SD))	327.59 (90.83)	327.40 (90.76)	377.47 (96.28)	<0.001
Creatinine (μmol/L, mean (SD))	72.93 (19.75)	72.88 (19.67)	86.02 (31.90)	<0.001
HGB (g/L, mean (SD))	144.23 (16.07)	144.23 (16.07)	146.03 (16.77)	<0.001
RDW-CV (%, mean (SD))	12.72 (1.15)	12.72 (1.14)	13.10 (1.22)	<0.001
WBC counts (×10^9^/L, mean (SD))	6.27 (1.75)	6.27 (1.75)	6.34 (1.71)	<0.001
PLT counts (×10^9^/L, mean (SD))	214.56 (60.00)	214.69 (59.98)	180.37 (56.49)	<0.001
**Comorbidities**				
Hypertension (%)	848,374 (30.2)	842,477 (30.1)	5,897 (55.4)	<0.001
T2DM (%)	248,334 (8.4)	246,228 (8.4)	2,106 (18.8)	<0.001
CHD (%)	59,529 (2.0)	57,546 (1.9)	1,983 (17.3)	<0.001
Overweight or obesity (%)	13,37,675 (56.0)	13,31,362 (55.9)	6,313 (68.1)	<0.001
Dyslipidaemia (%)	11,73,927 (41.7)	11,70,054 (41.7)	3,873 (34.8)	<0.001

BMI, body mass index; SBP, systolic blood pressure; DBP, diastolic blood pressure; FBG, fasting blood glucose; BUN, blood urea nitrogen; UA, uric acid; HGB, hemoglobin; RDW-CV, red cell distribution width-coefficient of variation; WBC, white blood cell; PLT, platelets; TC, total cholesterol; TG, triglycerides; LDL-C, low-density lipoprotein cholesterol; HDL-C, high-density lipoprotein cholesterol; ALT, alanine transaminase; AST, aspartate transaminase; CHD, coronary heart disease; T2DM, type 2 diabetes mellitus.

Compared to the non-AF group, the AF group was older and had significantly higher BMI, SBP, DBP, fasting blood glucose (FBG), BUN, UA, creatinine, hemoglobin (HGB), white blood cell (WBC), and aspartate transaminase (AST) levels. The proportions of males (72.3% vs. 59.4%, *p* < 0.001), hypertension (55.4% vs. 30.1%, *p* < 0.001), T2DM (18.8% vs. 8.4%, *p* < 0.001), CHD (17.3% vs. 1.9%, *p* < 0.001), and overweight or obesity (68.1% vs. 55.9%, *p* < 0.001) were significantly higher in the AF group than the non-AF group.

### 3.2. The prevalence of AF from nationwide health check-up centers

**Figure 1:**
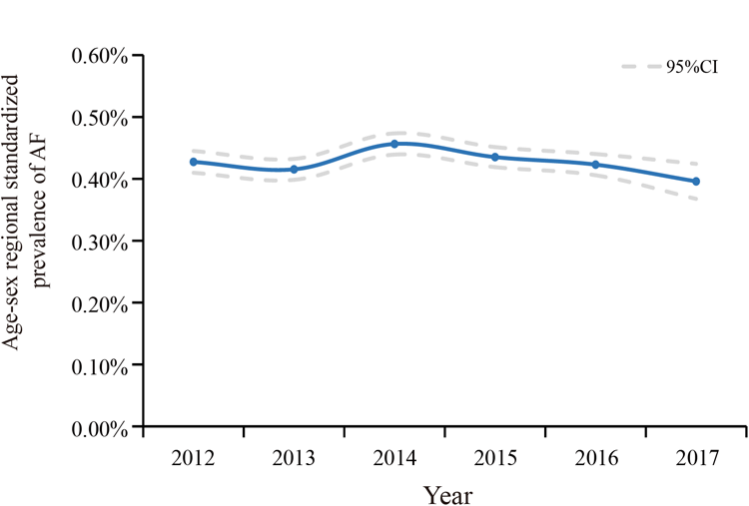
Temporal trend in age-, sex-, and regional-standardized prevalence of AF among health check-up population in China, 2012–2017. Calculating with specific weights based on the national population census of China in 2010.

**Figure 2:**
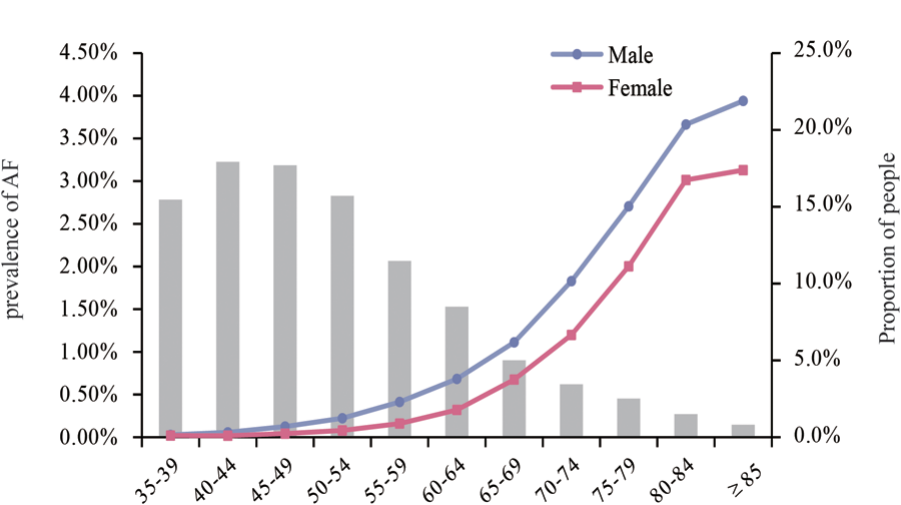
Prevalence of AF by age groups in males and females. And the proportion of the population in each age group.

The overall standardized prevalence estimate of AF was 0.43% (95%CI: 0.42%,0.44%) from 2012–2017. The change in prevalence was not significant throughout these years, with an APC −1.01 [95%CI: −4.25,2.33] (Figure 1; Table 2). In both sexes, the estimated prevalence of AF increased significantly with age. As shown in Figure 2, it ranges from less than 0.1% among participants aged 35–39 years to around 3.0% (female) and 4.0% (male) among participants aged 85 years or older. The estimated prevalence in the population over 65 years old (1.83%) was ten times higher than that in the 35–65-year age group (0.15%) (Table 3).

**Table 2:** Prevalence and trend of AF in health check-up population in China between 2012 and 2017.

	2012	2013	2014	2015	2016	2017	Overall	2012–2017 APC [95%CI]
	NO.	NO.	NO.	NO.	NO.	NO.	NO.	
	Prevalence	Prevalence	Prevalence	Prevalence	Prevalence	Prevalence	Prevalence	
	[95% CI]	[95% CI]	[95% CI]	[95% CI]	[95% CI]	[95% CI]	[95% CI]	
**Total** *								
	526,550	555,905	593,699	634,888	548,877	189,259	30,49,178	−1.01 [−4.25,2.33]
	0.43% [0.41%,0.45%]	0.42% [0.40%,0.43%]	0.46% [0.44%,0.47%]	0.44% [0.42%,0.45%]	0.42% [0.41%,0.44%]	0.40% [0.37%,0.42%]	0.43% [0.42%,0.44%]	
**Age groups** ^†^								
35–44	187,274	193,032	202,149	204,703	176,509	54,069	10,17,736	15.16 [6.42,24.62]
	0.02% [0.02%,0.03%]	0.03% [0.02%,0.04%]	0.03% [0.02%,0.03%]	0.03% [0.03%,0.04%]	0.04% [0.03%,0.04%]	0.05% [0.03%,0.07%]	0.03% [0.03%,0.03%]	
45–54	163,077	177,905	195,727	214,839	198,508	68,286	10,18,342	−1.72 [−7.88,4.86]
	0.14% [0.12%,0.16%]	0.11% [0.09%,0.12%]	0.11% [0.10%,0.13%]	0.11% [0.10%,0.12%]	0.12% [0.11%,0.14%]	0.12% [0.10%,0.15%]	0.12% [0.11%,0.12%]	
55–65	105,861	112,018	119,318	126,859	105,283	39,352	608,691	−3.31 [−7.76,1.36]
	0.40% [0.36%,0.44%]	0.38% [0.34%,0.42%]	0.39% [0.35%,0.42%]	0.40% [0.36%,0.43%]	0.37% [0.33%,0.40%]	0.31% [0.25%,0.36%]	0.38% [0.36%,0.39%]	
≥65	70,338	72,950	76,505	88,487	68,577	27,552	404,409	−1.01 [−5.57,3.77]
	1.67% [1.58%,1.77%]	1.70% [1.60%,1.79%]	1.88% [1.78%,1.98%]	1.80% [1.72%,1.89%]	1.69% [1.59%,1.79%]	1.57% [1.42%,1.71%]	1.74% [1.70%,1.78%]	
**Region** ^‡^								
** **Southern	316,764	314,070	344,211	360,716	322,381	76,993	17,35,135	2.34 [−1.27,6.09]
	0.39% [0.36%,0.41%]	0.36% [0.34%,0.38%]	0.43% [0.41%,0.45%]	0.43% [0.41%,0.45%]	0.42% [0.40%,0.44%]	0.42% [0.37%,0.47%]	0.41% [0.40%,0.42%]	
** **Northern	209,786	241,835	249,488	274,172	226,496	112,266	13,14,043	−4.88 [−8.93,−0.66]
	0.48% [0.45%,0.51%]	0.49% [0.46%,0.51%]	0.50% [0.47%,0.52%]	0.44% [0.42%,0.47%]	0.43% [0.40%,0.45%]	0.36% [0.33%,0.40%]	0.46% [0.45%,0.47%]	
**Sex** ^§^								
** **Male	314,250	330,362	355,224	374,781	327,002	109,814	18,11,433	−0.80 [−3.86,2.36]
	0.50% [0.48%,0.53%]	0.49% [0.46%,0.51%]	0.53% [0.50%,0.55%]	0.51% [0.49%,0.54%]	0.51% [0.48%,0.53%]	0.46% [0.42%,0.50%]	0.51% [0.50%,0.52%]	
** **Female	212,300	225,543	238,475	260,107	221,875	79,445	12,37,745	−1.31 [−5.24,2.78]
	0.35% [0.32%,0.37%]	0.34% [0.32%,0.37%]	0.38% [0.36%,0.41%]	0.35% [0.33%,0.38%]	0.34% [0.31%,0.36%]	0.33% [0.29%,0.37%]	0.35% [0.34%,0.36%]	

AF, atrial fibrillation; CI, confidence interval; APC, annual percentage change.

* Prevalence of AF in China was standardized by age, sex, and region.

^†^ Prevalence of AF in different age groups was standardized by sex and region.

^‡^ Prevalence of AF in southern and northern China was standardized by age and sex.

^§^ Prevalence of AF in male and female were standardized by age and region.

**Table 3:** Prevalence of AF in each disease subgroup.

Age	Both	Male	Female
	Prevalence	Prevalence	Prevalence
	[95% CI]	[95% CI]	[95% CI]
**Total population of this study**			
35–64	0.15% [0.15%,0.16%]	0.20% [0.20%,0.21%]	0.08% [0.08%,0.09%]
≥65	1.83% [1.79%,1.87%]	2.11% [2.06%,2.17%]	1.40% [1.35%,1.46%]
**Hypertension**			
35–64	0.27% [0.26%,0.29%]	0.31% [0.29%,0.33%]	0.18% [0.16%,0.20%]
≥65	2.09% [2.02%,2.15%]	2.37% [2.28%,2.45%]	1.65% [1.55%,1.74%]
**Type 2 diabetes** **mellitus**			
35–64	0.36% [0.33%,0.38%]	0.39% [0.35%,0.42%]	0.26% [0.21%,0.31%]
≥65	2.26% [2.14%,2.37%]	2.46% [2.31%,2.61%]	1.91% [1.74%,2.09%]
**Overweight or obesity (BMI ≥ 24 kg/m^2^)**			
35–64	0.22% [0.21%,0.23%]	0.25% [0.24%,0.26%]	0.13% [0.12%,0.14%]
≥65	2.19% [2.12%,2.26%]	2.47% [2.38%,2.56%]	1.75% [1.65%,1.85%]
**Coronary heart disease**			
35–64	1.25% [1.12%,1.39%]	1.38% [1.21%,1.54%]	0.92% [0.70%,1.14%]
≥65	5.08% [4.84%,5.32%]	5.78% [5.47%,6.10%]	3.80% [3.44%,4.15%]

AF, atrial fibrillation; BMI, body mass index.

**Figure 3:**
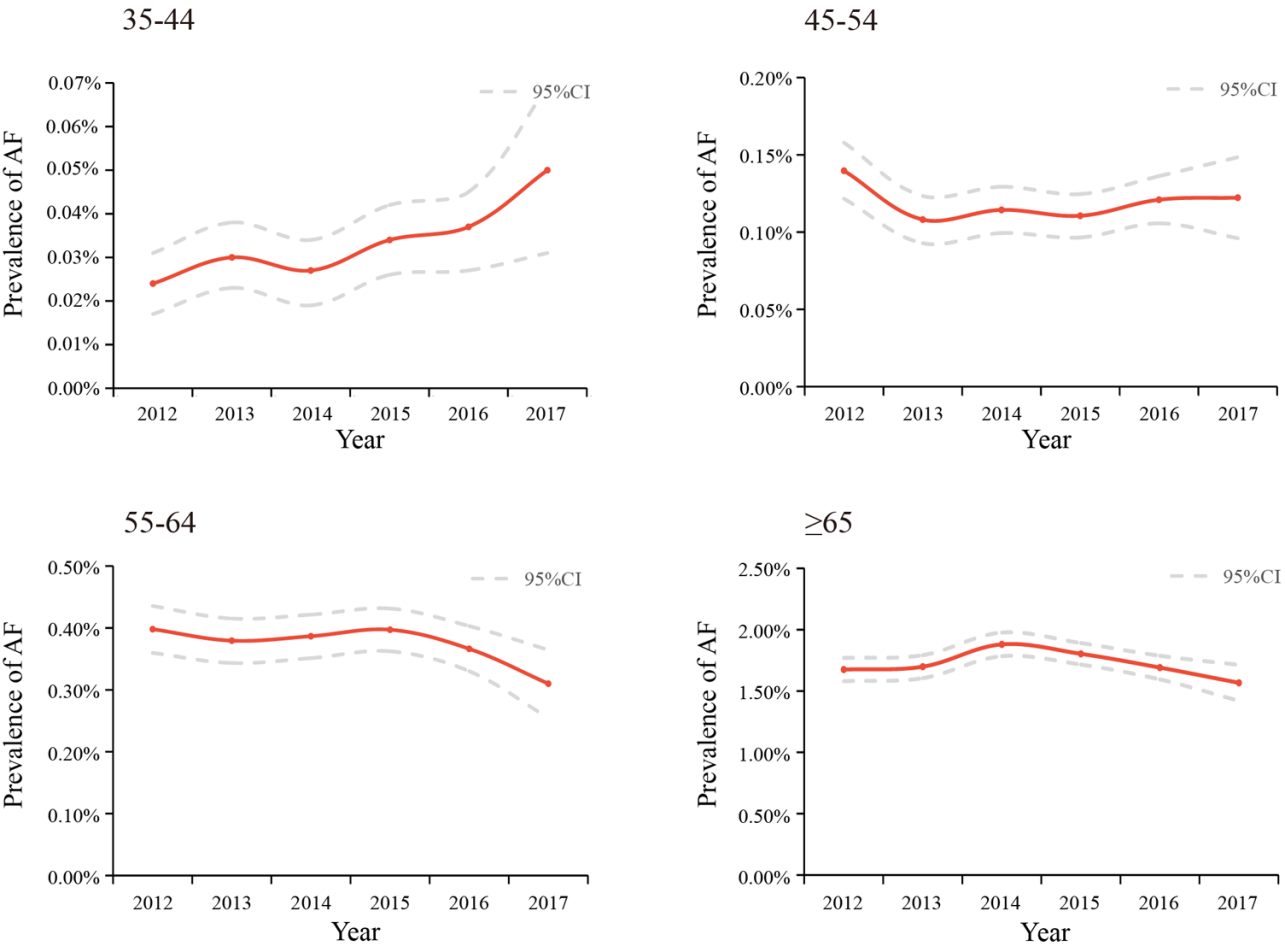
Temporal trends in the prevalence of AF among different age groups between 2012 and 2017, standardized by sex and region.

The trends of the estimated prevalence of AF among different age subgroups from 2012–2017 are presented in Table 2 and Figure 3. Remarkably, the prevalence of AF showed a significant ascending trend in the 35–44-year age group from 2012–2017, with an APC of 15.16 [95%CI: 6.42,24.62]. The prevalence of AF in the population aged 45–54 (APC: −1.72 [95%CI: −7.88,4.86]) and 55–64 (APC: −3.31 [95%CI: −7.76,1.36]) years old showed a stable trend. As for the population ≥65 years old, the prevalence of AF was also relatively stable, with an APC: of −1.01 [95%CI: −5.57,3.77], which was similar to the trend in the prevalence of AF in the overall population.

Between 2012 and 2017, the estimated prevalence of AF was higher in northern China than in southern China. The average prevalence of AF was 0.46% (95%CI: 0.45%,0.47%) in the north and 0.41% (95%CI: 0.40%,0.42%) in the south (Table 2). There was a slight decrease in northern China (APC: −4.88 [95%CI: −8.93,−0.66]) (Table 2, Supplementary Figure S1). In addition, in the sex subgroup, the prevalence of AF remained stable between 2012 and 2017 in both males and females. As shown in Table 3 and Supplementary Table S3, people with hypertension, T2DM, CHD, and overweight or obesity had a higher prevalence of AF. Among the 35–64-year age group, the prevalence of AF in people with hypertension, T2DM, overweight or obesity, and CHD was 0.27%, 0.36%, 0.22%, and 1.25%, respectively. In the group over 65, the prevalence of AF in people with hypertension, T2DM, overweight or obesity, and CHD was 2.09%, 2.26%, 2.19%, and 5.08%, respectively. Meanwhile, the prevalence of AF was significantly higher in males than females in all age groups and disease subgroups.

### 3.3. Risk factors associated with the prevalence of AF

**Figure 4:**
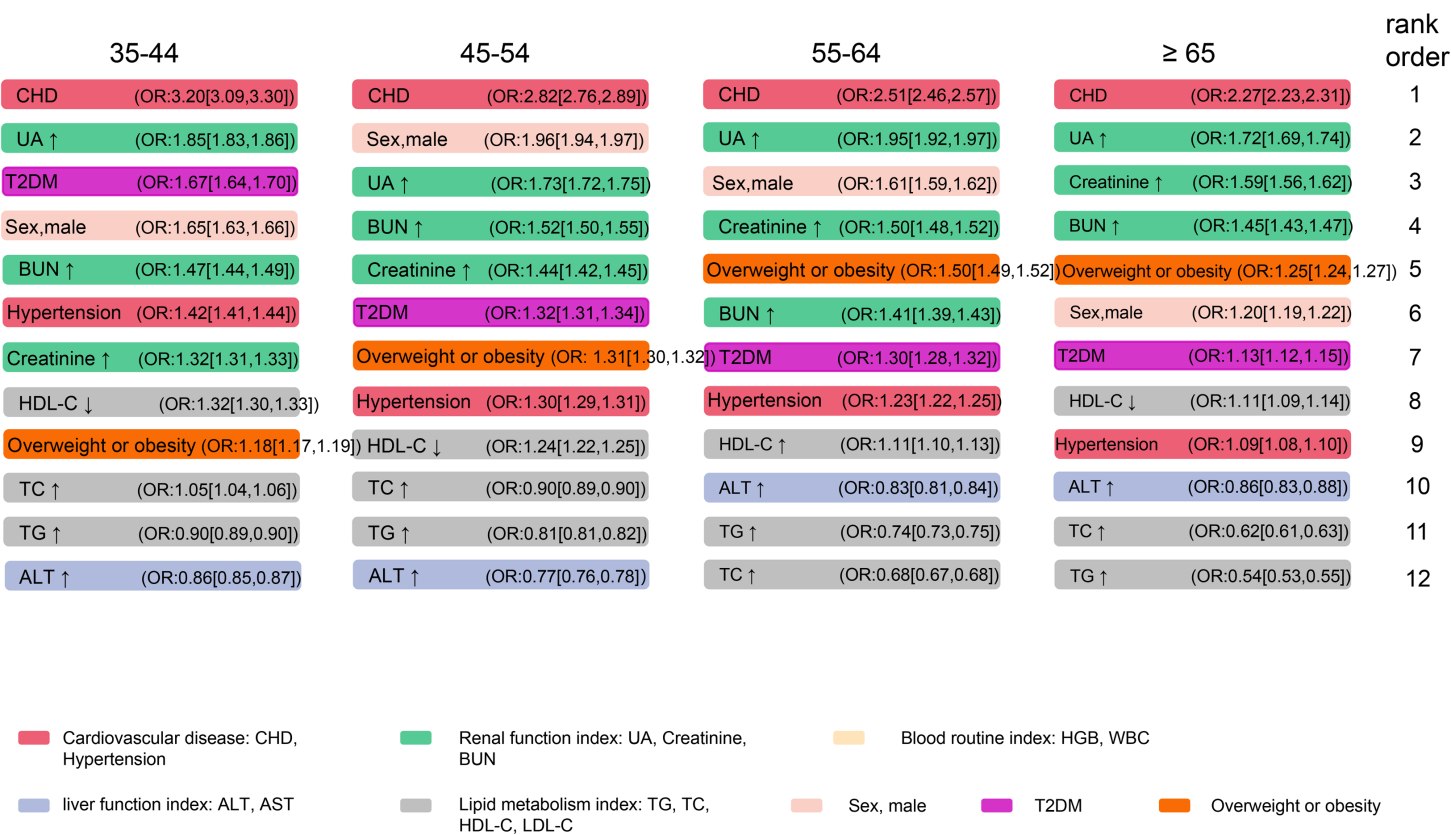
Associations between selected variables and AF among different age groups. The cut-off value for the index to rise or fall, and the abbreviations are consistent with Table 4. AF, atrial fibrillation; CHD, coronary heart disease; UA, uric acid; BUN, blood urea nitrogen; TC, total cholesterol; TG, triglycerides; HDL-C, high-density lipoprotein cholesterol; ALT, alanine transaminase; T2DM, type 2 diabetes mellitus.

Variables associated with the prevalence of AF were identified by Boruta algorithms, LASSO, and ranked by logistic regression models. These variables comprised sex, age, BMI, BUN, UA, creatinine, HDL-C, LDL-C, TG, TC, ALT, AST, HGB, WBC, CHD, T2DM, and hypertension. We revealed that age ≥65, CHD, elevated UA and BUN levels, T2DM, and hypertension were the leading factors for the prevalence of AF (Table 4). The rank of variables associated with AF varied among different age groups (Figure 4). CHD was the most significant variable in all age groups, followed by elevated UA in the 35–44-year, 55–64-year, and ≥65-year age groups. Besides, there was a strong correlation between elevated kidney function indexes (creatinine and BUN) and AF in all age groups. Interestingly, the rank of obesity or overweight gradually increased with age and surpassed that of hypertension and T2DM in the 55–64-year age group.

**Table 4:** Risk factors associated with AF by RF-boruta, LASSO regression model, and multivariate logistic regression model.

variable	Logistic's OR [95% CI]	*P*-value	LASSO's β	Importance in Boruta analysis
Age ≥ 65	6.46 [6.42,6.50]	<0.001	0.357	445.70(√)
CHD	2.85 [2.83,2.88]	<0.001	0.136	478.64(√)
UA (Male ≥ 420 μmol/L; Female ≥ 360 μmol/L)	1.83 [1.82,1.84]	<0.001	0.090	214.68(√)
BUN > 7.1 mmol/L	1.76 [1.75,1.78]	<0.001	0.077	229.91(√)
T2DM	1.51 [1.50,1.52]	<0.001	0.053	166.47(√)
Hypertension	1.51 [1.50,1.51]	<0.001	0.069	112.57(√)
Sex, male (%)	1.50 [1.49,1.51]	<0.001	0.059	87.27(√)
Creatinine elevated	1.40 [1.39,1.41]	<0.001	0.037	125.10(√)
Overweight or obesity (BMI ≥ 24 kg/m^2^)	1.39 [1.38,1.40]	<0.001	0.045	69.04(√)
HDL-C ≤ 1.0 mmol/L	1.17 [1.16,1.18]	<0.001	0.004	121.06(√)
TC ≥ 5.2 mmol/L	0.76 [0.75,0.76]	<0.001	−0.030	83.24(√)
ALT ≥ 40IU/L	0.66 [0.65,0.66]	<0.001	−0.042	68.08(√)
TG ≥ 1.7 mmol/L	0.65 [0.65,0.66]	<0.001	−0.055	110.57(√)
LDL-C ≥ 3.4 mmol/L			–	86.72(√)
HGB (Male ≤ 130 g/L; Female ≤ 115 g/L)			–	152.57(√)
WBC ≥ 10 × 10^9^/L			–	125.45(√)
AST ≥ 40IU/L			–	125.22(√)

AF, atrial fibrillation; CHD, coronary heart disease; UA, uric acid; BUN, blood urea nitrogen; HDL-C, high-density lipoprotein cholesterol; LDL-C, low-density lipoprotein cholesterol; TC, total cholesterol; TG, triglycerides; HGB, hemoglobin; WBC, white blood cell; ALT, alanine transaminase; AST, aspartate transaminase; BMI, body mass index; T2DM, type 2 diabetes mellitus; OR, odds ratio; CI, confidence interval.

√, and × coding according to the Boruta analysis result: √ = confirmed, ? = tentative, and ×  = rejected.

## 4. Discussion

This present study is the first to unravel the prevalence and temporal trends of AF among the health check-up population using the largest cross-sectional database of 3,049,178 individuals and explore potential risk factors associated with AF. The overall estimated prevalence of AF was 0.43% from 2012 to 2017 and showed a substantial augmentation of the age-related prevalence. Even if there was no significant upward trend in the overall population from 2012 to 2017, we observed an obvious increase in the prevalence of AF among people aged 35–45. In addition, the prevalence of AF was higher in northern China than in southern China. We explored risk factors for AF in the overall population and different age groups. In this cross-sectional study, we observed that age ≥65, CHD, and UA level were the leading factors associated with the overall prevalence of AF. Moreover, renal function seems to be another key contributor to AF. With the increase of age, the correlation between obesity and AF gradually exceeds that between T2DM, hypertension, and AF.

### 4.1. The prevalence and trend of AF

According to a study based on a national representative sample of 29,079 people, the prevalence rate of AF in China's general population (≥30 years) was 0.65% in 2008 (4). Additionally, a national study of 726,451 people using 2-stage stratified cluster sampling found that the prevalence of AF was 2.31% among people aged ≥40 in 2018 (6). In 2020, a study based on a random sample of people in Liaoning province showed that the prevalence of AF in people aged ≥ 40 years in northern China was 1.1% (28). In 2022, the most recent national-wide study using a multistage stratified sampling of 114,039 people reported that the prevalence of AF was 1.6% in the Chinese adult population (7). Compared with these studies, the overall prevalence of AF among people over 35 years of age in the national health check-up centers is relatively lower. A key reason for the lower prevalence rate is the characteristics of the physical examination population itself and the younger population. The physical examination population, which is mainly composed of the occupational population, has a relatively higher level of education and health awareness (29). On the other hand, a number of paroxysmal AF was underdiagnosed, which partly contributed to the lower prevalence of AF in this study. During physical examinations, a single 12-lead electrocardiogram as a screening tool for AF will be biased toward identifying non-paroxysmal AF. Thus, non-paroxysmal AF may be the majority of AF types in our study. And our findings are more likely to characterize the nationwide epidemiology of non-paroxysmal AF in the health check-up population. Despite these, the large sample size and multicenter design spanning six years allowed us to systematically analyze the prevalence and trend of AF and its risk factors in the nationwide health check-up population, providing important references for prevention and control. Consistent with previous studies, the prevalence of AF has a differentiated age distribution in our study (i.e., the prevalence in older adults is much higher than in relatively younger populations). The prevalence of AF in males and northern areas was higher than that in females and southern areas.

The overall standardized prevalence of AF is relatively stable, similar to the trend in prevalence among the elderly (≥65 years old), who were the main patients with AF. In recent years, improvements in awareness and control of hypertension and T2DM, which contribute to well-controlled blood sugar and blood pressure levels, might have curbed the progression of AF to some extent, especially in the elderly (30, 31). We should continue to focus on these improvements and make good use of the fact that the population undergoing physical examination is the main audience for primary health education and health intervention. In contrast, the prevalence of AF in people aged 35–44, while low, presented an undesirable increasing trend from 2012–2017. On the one hand, CHD, hypertension, and T2DM are becoming younger and more common in China partly contributing to this result (32–34). At the same time, no doubt the social characteristics of this population may also be relevant. People aged 35–45 tend to be at the peak of their careers, and they may bear a large burden from work. Frequent overtime, social engagements, and staying up late make them often overlook their health status and maintain an unhealthy lifestyle. Additionally, in China, this population is also under pressure from the family to raise children (usually a minor or even young children) and support their parents. Meanwhile, alternative explanations should also be considered. All these might contribute to the undesirable increasing trend in the prevalence of AF among this population and suggest an urgent need for more attention and efficient measures to improve their situation.

### 4.2. Exploration of risk factors

Under the background of the discovery of new energy and technological progress, the combination of surgical and new energy technologies has become mainstream, and the conversion rate of AF to sinus rhythm has also gradually increased (35, 36). However, the high recurrence rate in the long-term follow-up is still a challenge (35). Therefore, the management of risk factors for AF remains a cornerstone in the comprehensive prevention and treatment of AF. Among the variables analyzed, male sex, aging, overweight or obesity, hypertension, T2DM, and CHD have already been established and recognized as risk factors for AF (37). In the present study, we found that age ≥65 (OR: 6.46 [6.42,6.50]) was the most significant risk factor of AF overall among the health check-up population, followed by CHD (OR: 2.85 [2.83,2.88]) which was also consistently the most important risk factor in each age group (Table 4, Figure 4). In addition, as Figure 4 shows, the strength of the association between overweight or obesity and AF exceeds that between hypertension, T2DM, and AF with increasing age. These remind us that, in younger people, prevention and control of T2DM and hypertension may be more important for AF. However, in the elderly, BMI was worth well to be managed.

It is worth noting that elevated UA was significantly associated with AF in this population, with a risk ratio secondary to that of CHD in each age group. The accumulated evidence revealed that elevated UA and hyperuricemia are associated with an increased risk of AF (38). A study using Medicare data also reported that allopurinol, a urate-lowering drug, was associated with a reduced risk of incident AF (39). The elevated UA promotes the development of cardiovascular diseases via multiple mechanisms, including the inflammatory response, oxidative stress, and insulin resistance (40). Worryingly, with the prevalence and incidence of hyperuricemia in China rising, hyperuricemia has not been given sufficient attention it deserves (41, 42). This may also have contributed to the increased prevalence of AF in people aged 35–44. Whether controlling or attenuating the levels of UA prevents the development of AF remains to be validated in the prospective study.

Moreover, the elevated kidney function indexes (creatinine, BUN) were also strongly associated with an increased risk of AF in almost all age groups. A number of studies have reported that chronic kidney disease increases the risk of developing AF due to excessive extracellular fluid and toxins, and the activation of the renin-angiotensin-aldosterone system results in atrial remodeling (43, 44). Conversely, several studies also showed that AF increased the risk of the development of kidney disease (45). More recently, Sehoon Park et al. have made further progress in revealing the causal relationship between AF and renal function (46). According to the result of bidirectional Mendelian randomization analysis, AF is considered a causal risk factor for kidney function impairment, but an effect of kidney function on AF was not identified. The relationship between AF and CKD still needs to be further explored.

## 5. Limitation

Several limitations should be noted. First, the study population was not based on representative sampling; although age, sex, and region have been adjusted, selection bias is inevitable. Second, the detection of AF has primarily relied on a single 12-lead ECG in the office, not a wearable electrocardiogram or Holter. Paroxysmal AF could be underdiagnosed. Thus, our findings are more likely to characterize the nationwide epidemiology of non-paroxysmal AF in the health check-up population. Third, due to excessive omissions of information, some other risk factors and medication were not included in the risk factor analysis. Fourth, no doubt, variations in social and economic contexts may be relevant, but these data are also limited. Fifth, the missing values and imputations may also lead to an inevitable bias in the risk factor analysis. Sixth, the risk factor exploration in our cross-section study is correlational research. No cause and effect can be recognized. Seventh, the six-year study span may be limited, and a longer exploration period is needed to systematically explore the changing trends.

## 6. Conclusion

This large cross-sectional study comprehensively explored the prevalence and trend of AF among the health check-up population nationwide. Also, it explores the risk factors of AF in the overall population and different age groups. Although our results showed that the overall age-sex-and regional-standardized prevalence of AF among the health check-up population kept stable and fluctuated around 0.43% from 2012–2017, a great increased prevalence of AF in people aged 35–44 years was worrisome. For younger adults, hypertension and T2DM were associated with a higher risk of AF than obesity; while for older people, obesity was associated with a higher risk of AF than hypertension and T2DM. In addition to traditional leading risk factors, the tight correlations between elevated UA, impaired renal function, and AF have been brought to our attention.

## Data Availability

The raw data supporting the conclusions of this article will be made available by the authors, without undue reservation.

## References

[1] Zimetbaum P. Atrial fibrillation. Ann Intern Med. (2017) 166:ITC33–48. 10.7326/AITC201703070 28265666

[2] Wang L Ze F Li J Mi L Han B Niu H Trends of global burden of atrial fibrillation/flutter from global burden of disease study 2017. Heart. (2021) 107:881–7. 10.1136/heartjnl-2020-317656 33148545

[3] Dai H Zhang Q Much AA Maor E Segev A Beinart R Global, regional, and national prevalence, incidence, mortality, and risk factors for atrial fibrillation, 1990–2017: results from the global burden of disease study 2017. Eur Heart J Qual Care Clin Outcomes. (2021) 7:574–82. 10.1093/ehjqcco/qcaa061 32735316 PMC8557444

[4] Zhou Z Hu D. An epidemiological study on the prevalence of atrial fibrillation in the Chinese population of mainland China. J Epidemiol. (2008) 18:209–16. 10.2188/jea.je2008021 18776706 PMC4771592

[5] Li Y Wu YF Chen KP Li X Zhang X Xie GQ Prevalence of atrial fibrillation in China and its risk factors. Biomed Environ Sci. (2013) 26:709–16. 10.3967/0895-3988.2013.09.001 24099604

[6] Wang X Fu Q Song F Li W Yin X Yue W Prevalence of atrial fibrillation in different socioeconomic regions of China and its association with stroke: results from a national stroke screening survey. Int J Cardiol. (2018) 271:92–7. 10.1016/j.ijcard.2018.05.131 29885822

[7] Shi S Tang Y Zhao Q Yan H Yu B Zheng Q Prevalence and risk of atrial fibrillation in China: a national cross-sectional epidemiological study. Lancet Reg Health West Pac. (2022) 23:100439. 10.1016/j.lanwpc.2022.100439 35800039 PMC9252928

[8] Cao X Chen Z Wu L Zhou J. Co-occurrence of chronic pain, depressive symptoms, and poor sleep quality in a health check-up population in China:a multicenter survey. J Affect Disord. (2021) 281:792–8. 10.1016/j.jad.2020.11.060 33229026

[9] Fuster V Ryden LE Cannom DS Crijns HJ Curtis AB Ellenbogen KA ACC/AHA/ESC 2006 guidelines for the management of patients with atrial fibrillation: a report of the American college of cardiology/American heart association task force on practice guidelines and the European society of cardiology committee for practice guidelines (writing committee to revise the 2001 guidelines for the management of patients with atrial fibrillation): developed in collaboration with the European heart rhythm association and the heart rhythm society. Circulation. (2006) 114:e257–354. 10.1161/CIRCULATIONAHA.106.177292 16908781

[10] Kirchhof P Benussi S Kotecha D Ahlsson A Atar D Casadei B 2016 ESC guidelines for the management of atrial fibrillation developed in collaboration with EACTS. Eur Heart J. (2016) 37:2893–962. 10.1093/eurheartj/ehw210 27567408

[11] Chen C Lu FC. The guidelines for prevention and control of overweight and obesity in Chinese adults. Biomed Environ Sci. (2004) 26(17 Suppl):1–4. 15807475

[12] 2018 Chinese guidelines for prevention and treatment of hypertension-A report of the revision committee of Chinese guidelines for prevention and treatment of hypertension. J Geriatr Cardiol. (2019) 16:182–241. 10.11909/j.issn.1671-5411.2019.03.014 31080465 PMC6500570

[13] Chinese Diabetes Society. Guidelines for the prevention and treatment of type 2 diabetes mellitus in China (2020 edition). Chin J Diabetes Mellit. (2021) 13:315–409. 10.3760/cma.j.cn115791-20210221-00095

[14] Chinese Guidelines on prevention and treatment of dyslipidemia in adults. Zhonghua Xin Xue Guan Bing Za Zhi. (2007) 35:390–419. 17711682

[15] Chinese Kidney Society. Chinese Practice guidelines for diagnosis and treatment of hyperuricemia in renal disease (2017 edition). Zhonghua Yi Xue Za Zhi. (2017) 97:1927–36.

[16] Wang X Xu G Li H Liu Y Wang F. Reference intervals for serum creatinine with enzymatic assay and evaluation of four equations to estimate glomerular filtration rate in a healthy Chinese adult population. Clin Chim Acta. (2011) 412:1793–7. 10.1016/j.cca.2011.05.033 21672534

[17] Walker HK Hall WD Hurst JW, eds. Clinical methods: The history, physical, and laboratory examinations. Boston: Butterworth Publishers (1990) Butterworths Copyright © 1990, a division of Reed Publishing. 21250045

[18] Chinese Guidelines on prevention and treatment of dyslipidemia in adults (2016 revision). Chin Circulation J. (2016) 31:937–50. 10.3969/j.issn.1000-3614.2016.10.001

[19] Baldi I Gruber A Alioum A Berteaud E Lebailly P Huchet A Descriptive epidemiology of CNS tumors in France: results from the gironde registry for the period 2000–2007. Neuro Oncol. (2011) 13:1370–8. 10.1093/neuonc/nor120 21980160 PMC3223087

[20] Paik JM Golabi P Younossi Y Mishra A Younossi ZM. Changes in the global burden of chronic liver diseases from 2012 to 2017: the growing impact of NAFLD. Hepatology. (2020) 72:1605–16. 10.1002/hep.31173 32043613

[21] Stekhoven DJ Buhlmann P. Missforest–non-parametric missing value imputation for mixed-type data. Bioinformatics. (2012) 28:112–8. 10.1093/bioinformatics/btr597 22039212

[22] Wallentin L Eriksson N Olszowka M Grammer TB Hagström E Held C Plasma proteins associated with cardiovascular death in patients with chronic coronary heart disease: a retrospective study. PLoS Med. (2021) 18:e1003513. 10.1371/journal.pmed.1003513 33439866 PMC7817029

[23] Degenhardt F Seifert S Szymczak S. Evaluation of variable selection methods for random forests and omics data sets. Brief Bioinform. (2019) 20:492–503. 10.1093/bib/bbx124 29045534 PMC6433899

[24] Tibshirani R. Regression shrinkage and selection via the lasso. J R Stat Soc. (1996) 58:267–88. 10.1111/j.2517-6161.1996.tb02080.x

[25] Griffith KN Prentice JC Mohr DC Conlin PR. Predicting 5- and 10-year mortality risk in older adults with diabetes. Diabetes Care. (2020) 43:1724–31. 10.2337/dc19-1870 32669409 PMC7372062

[26] Lunardon N Menardi G Torelli N. ROSE: a package for binary imbalanced learning. R J. (2014) 6:79–89. 10.32614/RJ-2014-008

[27] O'Neill AC Yang D Roy M Sebastiampillai S Hofer SOP Xu W. Development and evaluation of a machine learning prediction model for flap failure in microvascular breast reconstruction. Ann Surg Oncol. (2020) 27:3466–75. 10.1245/s10434-020-08307-x 32152777

[28] Xing L Lin M Du Z Jing L Tian Y Yan H Epidemiology of atrial fibrillation in northeast China: a cross-sectional study, 2017–2019. Heart. (2020) 106:590–5. 10.1136/heartjnl-2019-315397 31611327

[29] Chinese Health Management Association. Expert consensus on screening and management of risk factors for cardiovascular diseases in the Chinese physical examination population. Chin J Health Manag. (2015) 9:398–412. 10.3760/cma.j.issn.1674-0815.2015.06.003

[30] Wu L He Y Jiang B Sun D Wang J Liu M Trends in prevalence, awareness, treatment and control of hypertension during 2001–2010 in an urban elderly population of China. PLoS One. (2015) 10:e0132814. 10.1371/journal.pone.0132814 26241049 PMC4524712

[31] Li MZ Su L Liang BY Tan JJ Chen Q Long JX Trends in prevalence, awareness, treatment, and control of diabetes mellitus in mainland China from 1979 to 2012. Int J Endocrinol. (2013) 2013:753150. 10.1155/2013/753150 24288530 PMC3830848

[32] Li D Zeng X Huang Y Lei H Li G Zhang N Increased risk of hypertension in young adults in southwest China: impact of the 2017 ACC/AHA high blood pressure guideline. Curr Hypertens Rep. (2019) 21:21. 10.1007/s11906-019-0926-y 30820764

[33] Ma RCW. Epidemiology of diabetes and diabetic complications in China. Diabetologia. (2018) 61:1249–60. 10.1007/s00125-018-4557-7 29392352

[34] Wang X Gao M Zhou S Wang J Liu F Tian F Trend in young coronary artery disease in China from 2010 to 2014: a retrospective study of young patients ≤45. BMC Cardiovasc Disord. (2017) 17:18. 10.1186/s12872-016-0458-1 28061763 PMC5219759

[35] Latchamsetty R Morady F. Atrial fibrillation ablation. Annu Rev Med. (2018) 69:53–63. 10.1146/annurev-med-041316-090015 28806148

[36] Yamane T. Catheter ablation of atrial fibrillation: current status and near future. J Cardiol. (2022) 80:22–7. 10.1016/j.jjcc.2022.02.005 35221152

[37] Andrade J Khairy P Dobrev D Nattel S. The clinical profile and pathophysiology of atrial fibrillation: relationships among clinical features, epidemiology, and mechanisms. Circ Res. (2014) 114:1453–68. 10.1161/CIRCRESAHA.114.303211 24763464

[38] Kuwabara M Niwa K Nishihara S Nishi Y Takahashi O Kario K Hyperuricemia is an independent competing risk factor for atrial fibrillation. Int J Cardiol. (2017) 231:137–42. 10.1016/j.ijcard.2016.11.268 27871785

[39] Singh JA Yu S. Allopurinol and the risk of atrial fibrillation in the elderly: a study using medicare data. Ann Rheum Dis. (2017) 76:72–8. 10.1136/annrheumdis-2015-209008 27165177

[40] Yu W Cheng JD. Uric acid and cardiovascular disease: an update from molecular mechanism to clinical perspective. Front Pharmacol. (2020) 11:582680. 10.3389/fphar.2020.582680 33304270 PMC7701250

[41] Shan R Ning Y Ma Y Gao X Zhou Z Jin C Incidence and risk factors of hyperuricemia among 2.5 million Chinese adults during the years 2017–2018. Int J Environ Res Public Health. (2021) 18:2360. 10.3390/ijerph18052360 33671018 PMC7957707

[42] Multidisciplinary Expert Task Force on H, Related D. Chinese Multidisciplinary expert consensus on the diagnosis and treatment of hyperuricemia and related diseases. Chin Med J. (2017) 130:2473–88. 10.4103/0366-6999.216416 29052570 PMC5684625

[43] Alonso A Lopez FL Matsushita K Loehr LR Agarwal SK Chen LY Chronic kidney disease is associated with the incidence of atrial fibrillation: the atherosclerosis risk in communities (ARIC) study. Circulation. (2011) 123:2946–53. 10.1161/CIRCULATIONAHA.111.020982 21646496 PMC3139978

[44] Ding WY Gupta D Wong CF Lip GYH. Pathophysiology of atrial fibrillation and chronic kidney disease. Cardiovasc Res. (2021) 117:1046–59. 10.1093/cvr/cvaa258 32871005

[45] Bansal N Fan D Hsu C-y Ordonez JD Marcus GM Go AS. Incident atrial fibrillation and risk of end-stage renal disease in adults with chronic kidney disease. Circulation. (2013) 127:569–74. 10.1161/CIRCULATIONAHA.112.123992 23275377 PMC3676734

[46] Park S Lee S Kim Y Lee Y Kang MW Kim K Atrial fibrillation and kidney function: a bidirectional Mendelian randomization study. Eur Heart J. (2021) 42:2816–23. 10.1093/eurheartj/ehab291 34023889

