# Supplementary material for: Prevalence and trend of atrial fibrillation and its associated risk factors among the population from nationwide health check-up centers in China, 2012–2017
Source: Front Cardiovasc Med. 2023 May 31;10:1151575. doi: 10.3389/fcvm.2023.1151575 (PMC10264614; doi:10.3389/fcvm.2023.1151575)
Supplement: Supplementary file 1 [file Table1.docx]

**SUPPLEMENTAL MATERIAL**

**Title: Prevalence and trend of atrial fibrillation and its associated risk factors among the population** **from nationwide Health Check-Up Centers in China，2012-2017**

**Table S1. Location, sample size and study period of each health management centers.**

| **Center** | **Area** | **Distribution** | **Beginning and end dates** | **Volumes of data** |
| --- | --- | --- | --- | --- |
| C1 | Beijing | Northern | 2012-2017 | 165054 |
| C2 | Beijing | Northern | 2012-2013 | 15895 |
| C3 | Hohhot in Inner Mongolia | Northern | 2012-2017 | 97232 |
| C4 | Jilin city in Jilin | Northern | 2015-2017 | 54210 |
| C5 | Jinan in Shandong | Northern | 2012-2015 | 68074 |
| C6 | Linfen in Shanxi | Northern | 2014-2017 | 49719 |
| C7 | Lanzhou in Gansu | Northern | 2012-2017 | 98674 |
| C8 | Shijiazhuang in Hebei | Northern | 2012-2017 | 168072 |
| C9 | Shijiazhuang in Hebei | Northern | 2012-2017 | 476903 |
| C10 | Shenyang in Liaoning | Northern | 2012-2017 | 50325 |
| C11 | Tianjin | Northern | 2014-2015 | 28218 |
| C12 | Zhenzhou in Henan | Northern | 2012-2016 | 41667 |
| C13 | Chongqing | Southern | 2012-2017 | 499137 |
| C14 | Changsha in Hunan | Southern | 2012-2017 | 350568 |
| C15 | Changsha in Hunan | Southern | 2012-2013 | 23428 |
| C16 | Guangzhou in Guangdong | Southern | 2015-2017 | 20256 |
| C17 | Zhanjiang in Guangdong | Southern | 2013-2017 | 30619 |
| C18 | Nanchang in Jiangxi | Southern | 2012-2017 | 23673 |
| C19 | Wuhan in Hubei | Southern | 2012-2016 | 116499 |
| C20 | Wuhan in Hubei | Southern | 2012-2017 | 92779 |
| C21 | Wuhan in Hubei | Southern | 2012-2016 | 122702 |
| C22 | Wuhan in Hubei | Southern | 2012-2016 | 83968 |
| C23 | Wuhan in Hubei | Southern | 2014-2016 | 58576 |
| C24 | Wuhan in Hubei | Southern | 2012-2016 | 103651 |
| C25 | Shiyan in Hubei | Southern | 2012-2016 | 48857 |
| C26 | Shiyan in Hubei | Southern | 2012-2017 | 132438 |
| C27 | Xiangyang in Hubei | Southern | 2015-2016 | 27984 |

**Table S2. The missing rates of variables included in risk factor analysis.**

| **Variables** | **Missing rate (%)** |  | **Variables** | **Missing rate (%)** |
| --- | --- | --- | --- | --- |
| Gender | 0.00 |  | TC | 7.67 |
| Age | 0.00 |  | HGB | 9.19 |
| BMI | 21.65 |  | WBC | 9.03 |
| BUN | 13.04 |  | ALT | 6.96 |
| UA | 11.03 |  | AST | 33.43 |
| Creatinine | 10.93 |  | Hypertension | 7.95 |
| LDL-C | 21.79 |  | T2DM | 3.43 |
| HDL-C | 21.74 |  | CHD | 0.00 |
| TG | 7.70 |  |  |  |

Abbreviations: BMI, body mass index; BUN, blood urea nitrogen; UA, uric acid; LDL-C, low-density lipoprotein cholesterol; HDL-C, high-density lipoprotein cholesterol; TG, triglycerides; TC, total cholesterol; HGB, hemoglobin; WBC, white blood cell; ALT, alanine transaminase; AST, aspartate transaminase; T2DM, type 2 diabetes mellitus; CHD, coronary heart disease.

**Table S3. Prevalence of AF in each disease subgroup further stratified by region.**

|  | **Southern** | | | |  | **Northern** | | |
| --- | --- | --- | --- | --- | --- | --- | --- | --- |
|  | **Age** | **Both** | **Male** | **Female** |  | **Both** | **Male** | **Female** |
|  |  | **Prevalence** | **Prevalence** | **Prevalence** |  | **Prevalence** | **Prevalence** | **Prevalence** |
|  |  | **[95% CI]** | **[95% CI]** | **[95% CI]** |  | **[95% CI]** | **[95% CI]** | **[95% CI]** |
| Total population of this study | | | | | | | | |
|  | 35-64 | 0.13% [0.12%,0.13%] | 0.17% [0.16%,0.18%] | 0.07% [0.06%,0.07%] |  | 0.19% [0.18%,0.20%] | 0.25% [0.23%,0.26%] | 0.11% [0.10%,0.12%] |
|  | ≥65 | 1.74% [1.69%,1.80%] | 2.04% [1.96%,2.11%] | 1.28% [1.20%,1.35%] |  | 1.95% [1.88%,2.02%] | 2.23% [2.14%,2.33%] | 1.57% [1.48%,1.66%] |
| Hypertension | | | | | | | | |
|  | 35-64 | 0.22% [0.21%,0.24%] | 0.26% [0.24%,0.28%] | 0.14% [0.11%,0.16%] |  | 0.32% [0.30%,0.34%] | 0.37% [0.34%,0.39%] | 0.22% [0.19%,0.25%] |
|  | ≥65 | 1.95% [1.87%,2.03%] | 2.27% [2.16%,2.38%] | 1.42% [1.30%,1.53%] |  | 2.26% [2.16%,2.37%] | 2.51% [2.37%,2.65%] | 1.92% [1.78%,2.06%] |
| Type 2 diabetes mellitus | | | | | | | | |
|  | 35-64 | 0.26% [0.23%,0.30%] | 0.29% [0.25%,0.33%] | 0.18% [0.12%,0.24%] |  | 0.44% [0.40%,0.48%] | 0.48% [0.43%,0.53%] | 0.32% [0.25%,0.39%] |
|  | ≥65 | 2.14% [1.98%,2.29%] | 2.44% [2.24%,2.64%] | 1.56% [1.33%,1.78%] |  | 2.39% [2.22%,2.56%] | 2.48% [2.25%,2.70%] | 2.26% [1.99%,2.52%] |
| Overweight or obesity (BMI≥24 kg/m^2^) | | | | | | | | |
|  | 35-64 | 0.17% [0.16%,0.18%] | 0.20% [0.19%,0.21%] | 0.09% [0.07%,0.10%] |  | 0.27% [0.25%,0.28%] | 0.31% [0.29%,0.33%] | 0.17% [0.15%,0.19%] |
|  | ≥65 | 2.04% [1.96%,2.13%] | 2.35% [2.23%,2.47%] | 1.51% [1.38%,1.64%] |  | 2.38% [2.27%,2.49%] | 2.63% [2.48%,2.78%] | 2.02% [1.87%,2.18%] |
| Coronary heart disease | | | | | | | | |
|  | 35-64 | 0.85% [0.67%,1.03%] | 0.85% [0.64%,1.06%] | 0.84% [0.47%,1.20%] |  | 1.48% [1.30%,1.66%] | 1.69% [1.46%,1.91%] | 0.96% [0.69%,1.23%] |
|  | ≥65 | 5.12% [4.75%,5.49%] | 6.07% [5.58%,6.55%] | 3.07% [2.55%,3.58%] |  | 5.06% [4.74%,5.37%] | 5.55% [5.14%,5.97%] | 4.24% [3.77%,4.71%] |
