# Supplementary figures and images for: Prevalence and trend of atrial fibrillation and its associated risk factors among the population from nationwide health check-up centers in China, 2012–2017
Source: Front Cardiovasc Med. 2023 May 31;10:1151575. doi: 10.3389/fcvm.2023.1151575 (PMC10264614; doi:10.3389/fcvm.2023.1151575)

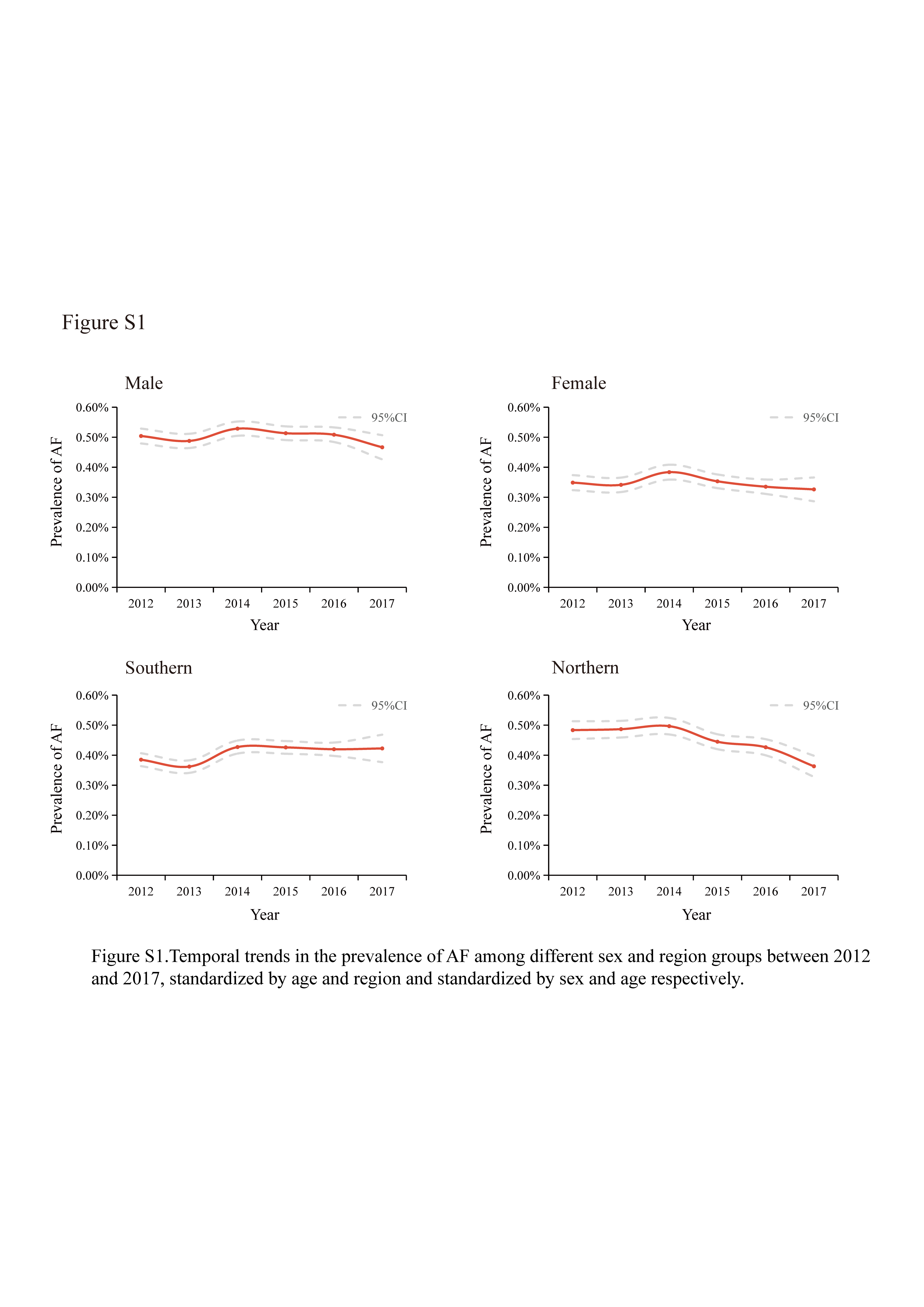

Supplement: Supplementary file 2 [file Image1.tif]
